# Women substance use in india: An important but often overlooked aspect

**DOI:** 10.1192/j.eurpsy.2021.2163

**Published:** 2021-08-13

**Authors:** R. Tripathi

**Affiliations:** Psychiatry, All India Institute of Medical Sciences, Gorakhpur, Gorakhpur, India

**Keywords:** women, Substance use, india, need

## Abstract

**Introduction:**

Substance abuse has traditionally been considered as a disease of men. Women were believed to have some kind of immunity in terms of “social inoculation”. However, due to change in societal norms and beliefs, substance use is currently increasing among women also.

**Objectives:**

To focus on female substance use in India

**Methods:**

In India, traditional use of various substances by women during religious festivals is not unknown. Chewing tobacco is a common practice among many women across the country. Cultural use of alcohol has been known in some tribal populations but gradually the use is increasing. There is major difference in pattern of male and female substance use including initiation, progression, recovery and relapse. Women experience greater medical, physiological and psychological impairment and experience loss of control sooner than males. Teatment needs of female substance users is different and requires a gender specific comprehensive strategy which will require medical services, mental health services, services for family and child and employment opportunities.

**Results:**

Currently, there is no Indian policy for women substance use. However, Government of India has started a convergence program which includes National AIDS Control program (NACP), National rural health mission (NRHM) and reproductive or sexually transmitted infection (RTI/STI) to combat some aspects.

**Conclusions:**

India is in great need of a policy or at least a standard operative protocol for management of female substance use disorder which may include screening for substance use disorder for all females accessing health sector, counselling, referral to addiction services, formation of a treating team and after –care.

**Disclosure:**

No significant relationships.

